# Risk Factors for Kidney Cancer in a Japanese Population: Findings from the JACC Study

**DOI:** 10.2188/jea.15.S203

**Published:** 2005-08-18

**Authors:** Masakazu Washio, Mitsuru Mori, Fumio Sakauchi, Yoshiyuki Watanabe, Kotaro Ozasa, Kyohei Hayashi, Tsuneharu Miki, Masahiro Nakao, Kazuya Mikami, Yoshinori Ito, Kenji Wakai, Akiko Tamakoshi

**Affiliations:** 1 Department of Public Health, Sapporo Medical University School of Medicine.; 2 Department of Epidemiology for Community Health and Medicine, Kyoto Prefectural University of Medicine.; 3 Department of Urology, Graduate School of Medical Sciences, Kyoto Prefectural University of Medicine.; 4 Department of Public Health, Fujita Health University School of Health Sciences.; 5 Division of Epidemiology and Prevention, Aichi Cancer Center Research Institute.; 6 Department of Preventive Medicine/Biostatistics and Medical Decision Making, Nagoya University Graduate School of Medicine.

**Keywords:** Kidney Neoplasms, Hypertension, Tea, fatty food, taro

## Abstract

BACKGROUND: The incidence of kidney cancer is high in Western and Northern Europe and North America, and low in Asia. Although the incidence of kidney cancer in Japan is lower than the rates in the other industrialized countries, there is no doubt that it is increasing.

METHODS: We evaluated the risk factors for kidney cancer death using the database of the Japan Collaborative Cohort (JACC) Study (i.e., medical history, anthropometry, and lifestyle including dietary habits). The analytic cohort included 47,997 males and 66,520 females aged 40 years and older. The Cox proportional hazards model was used to determine adjusted relative risks.

RESULTS: A total of 36 males and 12 females died from kidney cancer during the follow-up of 9.6 ± 2.6 years and 9.9 ± 2.2 years, respectively. A medical history of hypertension, a fondness for fatty food, and consumption of black tea were associated with an increased risk of kidney cancer death while an intake of taro, sweet potato and potato was associated with a decreased risk.

CONCLUSIONS: The present study showed four factors to be related to kidney cancer death. However, further studies may be needed to evaluate risk factors for kidney cancer death in Japan because the number of kidney cancer death in the present study was small.

Kidney cancer, which arises from cells of the proximal convoluted renal tubules,^1^^-^^3^ accounts for 2-3% of all malignancies in western countries^2^^-^^5^ and 1-2% in Japan.^5^^,^^6^ The incidence of kidney cancer is high in Western, and Northern Europe and North America whereas it is low in Asia.^1^^-^^3^ However, the incidence of kidney cancer is higher in Japanese Americans than in native Japanese.^5^ These findings suggest that environmental risk factors such as lifestyle factors may play an important role in the development of kidney cancer.

High-fat and high-protein diets,^7^ low physical activity,^8^^,^^9^ obesity,^1^^-^^3^^,^^10^^-^^13^ hypertension,^1^^-^^3^^,^^12^^-^^16^ kidney infections,^2^^,^^3^^,^^4^^,^^5^^,^^17^ kidney stones,^2^^,^^3^^,^^4^^,^^5^^,^^17^^,^^18^ and kidney cysts^2^^,^^3^^,^^4^^,^^5^^,^^17^ are reported to increase the risk of kidney cancer and many epidemiological reports exist on these area in western countries.^1^^-^^3^

The incidence and mortality of kidney cancer have been increasing in recent years in Japan,^6^ and from the viewpoint of prevention it is important to disclose the modifiable risk factors for this disease in Japan. However, relatively little information about the relationship between lifestyle and kidney cancer death in Japan is provided by a census-based cohort study.^19^

Therefore, in the present study, we evaluated the association between kidney cancer death and medical histories, body mass index and lifestyle factors (i.e., smoking and drinking status, dietary habits, and physical activity) on the basis of a large population-based cohort study in Japan (The Japan Collaborative Cohort Study for Evaluation of Cancer Risk [JACC] Study),^20^ which has been followed up for more than 1 million-person years.

## METHODS

The JACC Study, which is sponsored by the Ministry of Education, Culture, Sports, Science and Technology of Japan, is a nationwide collaborative prospective cohort study to evaluate the various risks and/or protective factors influencing cancer mortality and incidence.^20^ Study methods and ethical issues have been described elsewhere.^20^ Briefly, the cohort was established from 1988 to 1990, with 47,997 males and 66,520 females aged 40 years and older from 45 study areas across Japan. In the JACC Study, inhabitants aged 40 to 79 years at the baseline survey were usually used for the analyses. However, in this study, subjects aged 80 years and over were not excluded because the number of kidney cancer deaths was small (i.e., 48 cases). Most subjects were recruited from the general population or when undergoing routine health checks in the municipalities. The study subjects were followed up for mortality until the end of 1999. Written informed consent was obtained individually from participants, with the exception of a few study areas where informed consent was provided at the group level after explaining the aim of study and confidentiality of the data to community leaders. This investigation was approved by the Ethical Boards of Nagoya University School of Medicine and Kyoto Prefectural University of Medicine.

The self-administered questionnaire for the baseline survey included questions concerning medical history, height and weight, dietary habits of food and drink and lifestyle factors such as smoking, alcohol drinking, and physical activities.

Body mass index (BMI) was calculated as the weight divided by the square of height (kg/m^2^). BMI was categorized as low (BMI<18.5), intermediate (18.5 ≤ BMI<25.0), and high (BMI ≥ 25.0).

Population registries in the municipalities were used to determine vital and residential status of subjects. Registration of death is required by the Family Registration Law in Japan and is enforced throughout the country. The endpoint of the study was defined as death from kidney cancer (10th Revision of the International Statistical Classification of Diseases, ICD-10: C64). For logistical reasons, we discontinued the follow-up of subjects who moved out of their study area.

All statistical analyses were conducted using the Statistical Analysis System (SAS)^®^ package. The hazard ratios (HRs) of kidney cancer death and 95% confidence intervals (CIs) were estimated with Cox’s proportional hazard model. Age was treated as a continuous variable while indicator variables were used for other factors. The dose-dependent trend was tested by evaluating the regression coefficient when the three intake categories were treated as equally spaced numerical variables in Cox’s model. P values less than 0.05 were considered to be statistically significant.

## RESULTS

A total of 36 males and 12 females died from kidney cancer during the follow-up of 461, 066 and 658, 349 person-years, respectively. The mean (± standard deviation) follow-up periods were 9.6 ± 2.6 years for males and 9.9±2.2 years for females. Compared with females, males had a higher risk of kidney cancer death (age-adjusted HR = 4.63, 95% CI: 2.41-8.89), and the risk increased with age (sex-adjusted HR = 1.08 per 10-year increment, 95% CI: 1.05-1.11) (not shown in the table).

Table 1 shows the age- and sex-adjusted relative risk of kidney cancer death in relation to medical history. Hypertension was revealed as a significant risk factor for kidney cancer death (HR = 1.98, 95% CI: 1.06-3.70). Diabetes mellitus showed an HR greater than unity (HR = 2.28, 95% CI: 0.96-5.42), but was not a significant risk factor.

**Table 1.** Hazard ratio (HR) and 95% confidence interval (CI) for kidney cancer death according to medical history.

Medical history of disease		Person-years	No. of cases	Age-and sex-adjusted HR (95% CI)
Hypertension	(−)	776,575	24	1.00 (reference)
	(+)	224,177	18	1.98 (1.06-3.70)

Diabetes mellitus	(−)	923,005	37	1.00 (reference)
	(+)	50,708	6	2.28 (0.96-5.42)

Stroke	(−)	954,725	40	1.00 (reference)
	(+)	13,220	2	1.97(0.47-8.26)

Myocardial infarction	(−)	943,200	40	1.00 (reference)
	(+)	28,735	1	0.53 (0.07-3.89)

Kidney disease*	(−)	825,200	33	1.00 (reference)
	(+)	41,522	4	2.35 (0.83-6.64)

Liver disease	(−)	813,819	36	1.00 (reference)
	(+)	57,382	2	0.69 (0.17-2.85)

Cholecystitis or cholelithiasis	(−)	877,530	36	1.00 (reference)
	(+)	46,986	4	1.84 (0.65-5.19)

Tuberculosis or pleurisy	(−)	866,717	35	1.00 (reference)
	(+)	55,605	6	1.62 (0.67-3.88)

Cancer^†^	(−)	865,731	35	1.00 (reference)
	(+)	13,995	1	1.51 (0.21-11.12)

* : Kidney diseases other than kidney cancer.

† : Cancer other than kidney cancer.

As shown in Table 2, there was no meaningful association between body mass index and the risk of kidney cancer death. Table 3 illustrates the age- and sex-adjusted relative risk of kidney cancer death in relation to smoking status and drinking status. Compared with never-smokers, current smokers had an increased risk (HR = 2.13, 95% CI: 0.87-5.24), but the increase was not statistically significant. On the other hand, drinking status showed no meaningful relation to kidney cancer death.

**Table 2.** Hazard ratio (HR) and 95% confidence interval (CI) for kidney cancer death according to body mass index.

Body mass index (BMI, kg/m^2^)	Person-years	No. of cases	Age-and sex-adjusted HR (95% CI)	p for trend
BMI at baseline				
-18.4	62,200	2	0.59(0.140-2.47)	
18.5-25.0	767,730	31	1.00(reference)	
25.0+	221,127	11	1.51(0.76-3.02)	0.222

BMI at 20 years old				
-18.4	55,485	1	0.49(0.07-3.59)	
18.5-25.0	586,031	24	1.00(reference)	
25.0+	110,615	6	1.22(0.50-3.00)	0.406

**Table 3.** Hazard ratio (HR) and 95% confidence interval (CI) for kidney cancer death according to smoking and alcohol drinking status.

	Person-years	No. of cases	Age-and sex-adjusted HR (95% CI)	p for trend
Smoking status				
Never smokers	619,928	14	1.00(reference)	
Ex-smokers	125,091	9	1.36(0.48-3.82)	
Current smokers	262,030	21	2.13(0.87-5.24)	0.074

Alcohol drinking status				
Never drinkers	555,278	18	1.00(reference)	
Occational drinkers	200,640	6	0.86(0.34-2.22)	
Current drinkers^*^	250,268	20	1.42(0.69-2.90)	0.388

*: drink every day.

Table 4 shows HRs for kidney cancer death in relation to fondness for salty food and intake frequency of rice, miso soup, tofu, fresh fish, dried and salted fish, and pickles. There was no significant positive or negative association between these dietary factors and the risk of kidney cancer death.

**Table 4.** Hazard ratio (HR) and 95% confidence interval (CI) for kidney cancer death according to fondness for salty food and intake frequency of rice, miso soup, tofu, fish and pickles.

Dietary factors	Person-years	No. of cases	Age-and sex-adjusted HR (95% CI)	p for trend
Fond of salty food				
No	814,724	36	1.00(reference)	
Yes	75,282	5	1.45(0.57-3.70)	

Eat rice				
0-2 bowls/day	305,293	13	1.00(reference)	
3-5 bowls/day	667,071	28	0.99(0.51-1.92)	
6+ bowls/day	97,203	5	1.01(0.36-2.89)	0.992

Miso soup				
Seldom/some times	144,276	7	1.00(reference)	
Every other day	136,569	6	1.02(0.34-3.06)	
Every day	713,400	33	0.85(0.38-1.93)	0.633

Tofu(Soy bean curd)				
1-2/month or less	60,256	4	1.00(reference)	
1-4/week	261,343	10	0.62(0.20-1.99)	
Almost every day	611,260	32	0.86(0.30-2.43)	0.766

Fresh fish				
1-2/month or less	76,612	3	1.00(reference)	
1-4/week	608,019	25	1.13(0.34-3.74)	
Almost every day	248,412	14	1.49(0.43-5.21)	0.378

Dried and salted fish				
1-2/month or less	260,201	8	1.00(reference)	
1-4/week	297,280	13	1.43(0.59-3.45)	
Almost every day	223,994	13	1.83(0.76-4.41)	0.178

Pickles(Salted vegetables)				
1-2/month or less	99,834	3	1.00(reference)	
1-4/week	114,747	8	2.52(0.67-9.51)	
Almost every day	775,493	33	1.51(0.64-4.93)	0.927

Table 5 presents HRs for kidney cancer death in relation to fondness for fatty food and intake frequency of meats and dairy products. A fondness for fatty food was associated with a significantly increased risk (HR = 2.64, 95% CI: 1.03-6.78). Beef intake was positively (although not significantly; p for trend = 0.084) associated with risk of kidney cancer death. On the other hand, there was no meaningful association of other meat or dairy product consumption with kidney cancer death.

**Table 5.** Hazard ratio (HR) and 95% confidence interval (CI) for kidney cancer death according to fondness for fatty food and intake frequency of meats and dairy products.

Dietary factors	Person-years	No. of cases	Age-and sex-adjusted HR (95% CI)	p for trend
Fond of fatty food				
No	861,147	37	1.00(reference)	
Yes	37,962	5	2.64(1.03-6.78)	

Beef				
Seldom	185,171	7	1.00(reference)	
1-2/month	247,583	6	0.59(0.20-1.76)	
1-2/week +	291,879	21	1.73(0.74-4.08)	0.084

Pork				
Seldom	262,098	14	1.00(reference)	
1-2/month	354,109	14	0.84(0.40-1.77)	
1-2/week +	165,385	7	0.92(0.34-2.27)	0.785

Chicken				
1-2/month or less	288,490	11	1.00(reference)	
1-2/week	385,509	16	1.21(0.56-2.60)	
3-4/week +	171,960	10	1.62(0.69-3.81)	0.280

Ham and sausage				
Seldom	422,078	14	1.00(reference)	
1-2/month	297,969	14	1.67(0.79-3.51)	
1-2/week +	150,825	5	1.16(0.42-3.24)	0.488

Butter				
Seldom	387,814	15	1.00(reference)	
1-2/month	179,563	5	0.84(0.31-2.33)	
1-2/week +	191,373	10	1.47(0.66-3.24)	0.403

Cheese				
Seldom	405,245	16	1.00(reference)	
1-2/month	201,391	4	0.58(0.19-1.75)	
1-2/week +	159,199	7	1.14(0.47-2.78)	0.959

Milk				
1-2/month or less	264,742	12	1.00(reference)	
1-4/week	280,646	16	1.43(0.68-3.02)	
Almost every day	430,734	15	0.75(0.35-1.61)	0.375

Table 6 illustrates HRs for kidney cancer death in relation to intake frequency of vegetables and green tea, black tea and coffee. Starchy roots (i.e., taro, sweet potato and potato) reduced the risk of cancer death. Compared with 1-2 times/month or less, HRs were 0.67 (95% CI: 0.31-1.41) for 1-2 times/week and 0.44 (95% CI: 0.21-0.94) for 3-4 times/week or more. An intake of carrots and squash marginally decreased the risk of kidney cancer death (p for trend = 0.072). Compared with those who did not drink black tea, HRs were 2.15 (95% CI: 1.11-4.14) for those who drank 2 cups or less /day and 13.6 (95% CI: 1.83-101.30) for those who drank 3 cups or more /day. A non-significant increased risk was observed among coffee drinkers. Compared with nondrinkers, HRs were 1.25 (95% CI: 0.67-2.33) for those who drank 2 cups or less /day and 2.69 (95% CI: 0.89-8.10) for those who drank 3 cups or more /day.

**Table 6.** Hazard ratio (HR) and 95% confidence interval (CI) for kidney cancer death according to intake frequency of vegetables, green tea, black tea and coffee.

Dietary factors	Person-years	No. of cases	Age-and sex-adjusted HR (95% CI)	p for trend
Green-leafy vegetables				
1-2/month or less	74,207	4	1.00(reference)	
1-2/week	247,194	13	1.01(0.33-3.09)	
3-4/week +	540,633	21	0.70(0.24-2.03)	0.302

Carrots and squash				
1-2/month or less	136,675	9	1.00(reference)	
1-2/week	275,401	16	0.97(0.43-2.20)	
3-4/week +	383,789	14	0.64(0.33-1.45)	0.072

Chinese cabbage				
1-2/month or less	154,050	7	1.00(reference)	
1-2/week	445,064	23	1.00(0.43-2.34)	
3-4/week +	134,820	7	0.94(0.33-2.67)	0.905

Taro,sweet potato, and potato				
1-2/month or less	160,035	12	1.00(reference)	
1-2/week	338,804	16	0.67(0.31-1.41)	
3-4/week +	489,107	16	0.44(0.21-0.94)	0.034

Green tea				
None	72,658	6	1.00(reference)	
1-9 cups/day	632,686	24	0.87(0.44-1.69)	
10 + cups/day	75,444	6	1.52(0.58-4.00)	0.692

Black tea				
None	633,197	21	1.00(reference)	
2 cups/day or less	230,729	15	2.15(1.11-4.14)	
3+ cups/day	1,668	1	13.60(1.83-101.30)	0.004

Coffee				
None	245,022	8	1.00(reference)	
2 cups/day or less	506,494	21	1.25(0.67-2.33)	
3+ cups/day	57,643	4	2.69(0.89-8.10)	0.082

Table 7 shows the age- and sex-adjusted relative risk of kidney cancer death in relation to physical activity. There was no meaningful association between physical activity and kidney cancer death.

**Table 7.** Hazard ratio (HR) and 95% confidence interval (CI) for kidney cancer death according to physical activity.

Physical activity		Person-years	No. of cases	Age-and sex-adjusted HR (95% CI)
Leisure time physical activity				
	Physical exercise			
	Less than once a week	682,414	30	1.00(reference)
	Once a week or more	231,103	8	0.54(0.25-1.18)

	Walking			
	Less than 30 min/day	241,305	14	1.00(reference)
	30 min/day or more	571,389	23	0.69(0.36-1.34)

Occupational physical activity				
	Sedentary	360,189	14	1.00(reference)
	Active	395,302	19	1.44(0.72-2.88)

## DISCUSSION

Although the incidence of kidney cancer is lower in Japan than in other industrialized countries,^1^^-^^3^^,^^5^ there is no doubt that it has been increasing.^6^ This may be partly due to changes in lifestyle after World War II in Japan. The changed lifestyle in terms of westernized dietary habits, the spread of privately-owned cars and household electric appliances, and agricultural mechanization, may have increased the prevalence of obesity, by increasing animal protein and fat intake as well as by decreasing physical activity. Obesity^1^^-^^3^^,^^10^^-^^13^ and low physical activity^8^^,^^9^ are established risk factors for kidney cancer in western countries. However, in the present study, neither body mass index nor physical activity showed any significant relation to kidney cancer death in Japan.

In the present study, those who drank black tea had an increased risk of kidney cancer death even after adjusting for other factors. In addition, those who drank coffee had a marginally increased risk. However, there has been no convincing evidence linking kidney cancer and consumption of black tea or coffee despite numerous studies in western countries.^3^^,^^21^ In Japan, drinking black tea or coffee may be a surrogate for westernized dietary habits and thus it may be the latter rather than the former that is actually responsible for kidney cancer. Further studies are needed to ascertain whether there is any truth to this hypothesis.

Handa et al.^7^ reported that both a ‘dessert’ diet factor and a ‘beef’ diet factor were associated with an increased risk of kidney cancer, suggesting that high-fat and high-protein diets as well as sugar- and fat-rich confectioneries might be risk factors for kidney cancer. In the present study, fondness for fatty food as well as drinking black tea was associated with an increased risk.

Because the incidence of kidney cancer is higher in Japanese Americans than in native Japanese^5^ and it is increasing in Japan now,^6^ we cannot deny that westernization of dietary habits may play some role in the increased incidence of kidney cancer in Japan.

Chow et al.^22^ also reported that an intake of staple food (i.e., bread, cereals, potatoes, rice, and spaghetti) was associated with an increased risk of kidney cancer. On the other hand, Mucci et al.^23^ reported that none of potato, bread and cereal was a risk factor for kidney cancer. In the present study, an intake of starchy roots (i.e., taro, sweet potato and potato) was associated with a decreased risk of kidney cancer death while an intake of rice showed no meaningful relation. Taro^24^^,^^25^ and sweet potato,^26^ a part of the traditional Japanese diet, are reported to have cancer preventive potential, suggesting that these traditional diets may partly be the reasons for the lower incidence of kidney cancer death in Japan compared with the other developed countries.

Hypertension^12^^-^^16^^,^^27^ as well as anti-hypertensive medication^12^^,^^14^^-^^16^ has been reported to be a risk factor of kidney cancer. However, epidemiologic studies have not been able to distinguish the effects of hypertension from those of anti-hypertensive medications on the risk of kidney cancer.^13^ In the present study, a medical history of hypertension was associated with an increased risk of kidney cancer death.

Wideroff et al.^28^ found that diabetes mellitus was a risk factor for kidney cancer while Mellemgaard et al.^16^ did not. In the present study, a history of diabetes mellitus was marginally associated with an increased risk of kidney cancer death.

Kidney infections,^2^^,^^3^^,^^4^^,^^5^^,^^17^ kidney stones,^2^^,^^3^^,^^4^^,^^5^^,^^17^^,^^18^ and kidney cysts^2^^,^^3^^,^^4^^,^^5^^,^^17^ are risk factors for kidney cancer in western countries. However, in the present study, none of them was associated with an increased risk of kidney cancer death.

There are some limitations to our study. First, the number of kidney cancer deaths was very small in spite of the large scale of the study because of the small incidence of kidney cancer in Japan.^5^ Second, although male gender is an established risk factor for kidney cancer,^1^^-^^3^ we did not evaluate kidney cancer risk in men and women separately. Last, the endpoint of the present study was not the incidence of kidney cancer but kidney cancer death. In Japan, many cases of kidney cancer have been detected with renal imaging techniques such as ultrasonography.^6^ Thus, we cannot categorically deny that we may have missed an important risk factor for kidney cancer in Japan.

On the other hand, our study has its strengths as well. As far as we know, this is the first report on the risk factors for kidney cancer to evaluate lifestyle and medical histories in a large prospective study in the Japanese population.

In summary, the present study showed that hypertension, fondness for fatty food and drinking black tea were risk factors for kidney cancer death while a frequent intake of taro, sweet potato and potato was a preventive factor. However, these findings must be interpreted with caution. We could not evaluate kidney cancer risk in men and women separately because the number of kidney cancer deaths was small in the present study. Further studies may be needed to evaluate risk factors for kidney cancer death in Japan.

## MEMBER LIST OF THE JACC STUDY GROUP

The present investigators involved, with the co-authorship of this paper, in the JACC Study and their affiliations are as follows: Dr. Akiko Tamakoshi (present chairman of the study group), Nagoya University Graduate School of Medicine; Dr. Mitsuru Mori, Sapporo Medical University School of Medicine; Dr. Yutaka Motohashi, Akita University School of Medicine; Dr. Ichiro Tsuji, Tohoku University Graduate School of Medicine; Dr. Yosikazu Nakamura, Jichi Medical School; Dr. Hiroyasu Iso, Institute of Community Medicine, University of Tsukuba; Dr. Haruo Mikami, Chiba Cancer Center; Dr. Yutaka Inaba, Juntendo University School of Medicine; Dr. Yoshiharu Hoshiyama, Showa University School of Medicine; Dr. Hiroshi Suzuki, Niigata University School of Medicine; Dr. Hiroyuki Shimizu, Gifu University School of Medicine; Dr. Hideaki Toyoshima, Nagoya University Graduate School of Medicine; Dr. Shinkan Tokudome, Nagoya City University Graduate School of Medical Science; Dr. Yoshinori Ito, Fujita Health University School of Health Sciences; Dr. Shuji Hashimoto, Fujita Health University School of Medicine; Dr. Shogo Kikuchi, Aichi Medical University School of Medicine; Dr. Akio Koizumi, Graduate School of Medicine and Faculty of Medicine, Kyoto University; Dr. Takashi Kawamura, Kyoto University Center for Student Health; Dr. Yoshiyuki Watanabe, Kyoto Prefectural University of Medicine Graduate School of Medical Science; Dr. Tsuneharu Miki, Kyoto Prefectural University of Medicine Graduate School of Medical Science; Dr. Chigusa Date, Faculty of Human Environmental Sciences, Mukogawa Women’s University ; Dr. Kiyomi Sakata, Wakayama Medical University; Dr. Takayuki Nose, Tottori University Faculty of Medicine; Dr. Norihiko Hayakawa, Research Institute for Radiation Biology and Medicine, Hiroshima University; Dr. Takesumi Yoshimura, Institute of Industrial Ecological Sciences, University of Occupational and Environmental Health, Japan; Dr. Akira Shibata, Kurume University School of Medicine; Dr. Naoyuki Okamoto, Kanagawa Cancer Center; Dr. Hideo Shio, Moriyama Municipal Hospital; Dr. Yoshiyuki Ohno, Asahi Rosai Hospital; Dr. Tomoyuki Kitagawa, Cancer Institute of the Japanese Foundation for Cancer Research; Dr. Toshio Kuroki, Gifu University; and Dr. Kazuo Tajima, Aichi Cancer Center Research Institute.
